# Comparison between thermal ablation and surgery in low risk papillary thyroid carcinoma: a prospective study

**DOI:** 10.3389/fendo.2024.1398208

**Published:** 2024-08-01

**Authors:** Wenbo Gong, Runfang Zhang, Songtao Zhang, Yifei Zhai, Chen Zheng, Dongyan Zhang

**Affiliations:** Department of Thyroid and Head & Neck Surgery, The Affiliated Cancer Hospital of Zhengzhou University & Henan Cancer Hospital, Zhengzhou, China

**Keywords:** radiofrequency ablation, laser ablation, quality of life, papillary thyroid carcinoma, thermal ablation

## Abstract

**Objective:**

To conduct a comparative analysis of the efficacy, safety, and impact on quality of life outcomes between thermal ablation and surgical interventions in patients diagnosed with papillary thyroid carcinoma (PTC).

**Methods:**

A prospective study was undertaken, enrolling patients with PTC ≤5mm who underwent radiofrequency ablation (RFA), laser ablation (LA), or surgery, for analysis of efficacy and safety outcomes. The Thyroid Cancer-Specific Quality of Life questionnaire was administered to all patients before treatment and at 3, 6, and 12 months post-treatment.

**Results:**

A total of 162 eligible patients were included in the study. Major complications were not observed in the RFA and LA groups, while five cases were reported in the surgery group, although no statistically significant differences were observed. Minor complications were documented in two, three, and 14 patients in the RFA, LA, and surgery groups, respectively, with no significant variances noted. Surgical duration and hospitalization time were notably shorter in the thermal ablation groups. At the final follow-up, complete disappearance of nodules was seen in 71.4% of cases treated with RFA and 71.0% of cases managed with LA, with no significant disparities between the groups. Both RFA and LA exhibited similar effects on quality of life, with thermal ablation techniques showing better functional outcomes in comparison to surgery. Across all groups, adverse effects were most pronounced at the 3-month post-treatment mark but gradually reverted to baseline levels in the thermal ablation group, contrasting with the surgery group.

**Conclusions:**

For PTC ≤5mm, both RFA and LA exhibited similar cancer control outcomes and superior quality of life on par with surgery, while minimizing complications. These findings underscore the promise of RFA and LA as potential standard treatments for small PTCs, subject to further confirmation in future studies.

## Introduction

Papillary thyroid carcinoma (PTC) stands as the most predominant form of endocrine malignancy (1). Advances in health consciousness and the utilization of high-resolution ultrasound have led to the identification of an increasing number of PTC cases with a primary tumor diameter ≤1.0 cm and no discernible cervical lymph node enlargement (2). While surgery has historically been the favored treatment modality, the swift progression of thermal techniques, in which radiofrequency ablation (RFA) and laser ablation (LA) is the most frequently used, offers an alternative approach, especially for patients at risk of anesthesia complications or adverse to visible scarring (3).

Various investigations have explored the efficacy and safety of thermal ablation in PTC, highlighting its high feasibility, favorable rate of tumor resolution, effective local disease management, and minimal complications (4–6). Additionally, multiple meta-analyses have revealed that, in comparison to surgery, thermal ablation yields comparable quality of life (QoL) outcomes and non-inferior prognoses at a reduced cost (7–11). Despite these encouraging results, many of these studies are constrained by their retrospective nature, and the comparison of QoL between thermal ablation and endoscopic surgery remains equivocal. Furthermore, occult metastasis plays a crucial role in determining treatment strategies, as delayed detection of occult metastasis can lead to increased surgical complications. Unfortunately, this aspect has often been overlooked in previous studies. It is worth noting that occult metastasis can develop in over 50% of cases of cT1N0 PTC (12), with tumor size playing a significant role. Current evidence indicates that compared to PTC ≤5mm, the risk of lymph node metastasis is nearly five times higher in cases of PTC >5mm (13).

Henceforth, our aim is to undertake a prospective assessment of the effectiveness, safety, and QoL outcomes of thermal ablation and endoscopic surgery in individuals diagnosed with cN0 PTC ≤5mm.

## Patients and methods

### Ethical considerations

This study was approved by the Institutional Research Committee of Henan Cancer Hospital (No.2020-LC156), and all patients provided written informed consent for participation in medical research prior to undergoing treatment. All methods and procedures adhered to relevant guidelines and regulations.

### Study design

A prospective investigation was carried out from January 2021 to January 2022 to explore the favored therapeutic approach among individuals with cN0 solitary PTC ≤5mm. Prior to treatment, all patients underwent preoperative fine needle aspiration biopsy and genetic mutation profiling encompassing BRAF, RAS, RET, CTNNB1, TERT, PAX8-PPARγ, and PTEN. The findings were evaluated in accordance with the Bethesda classification system (14), with only patients exhibiting thyroid nodules categorized as Bethesda category V or VI being included in the study. In instances where two or more gene mutations were identified, surgery was recommended over thermal ablation. The selection of treatment modality predominantly hinged upon patient preference and the proficiency of the surgical team.

**Figure 1:**
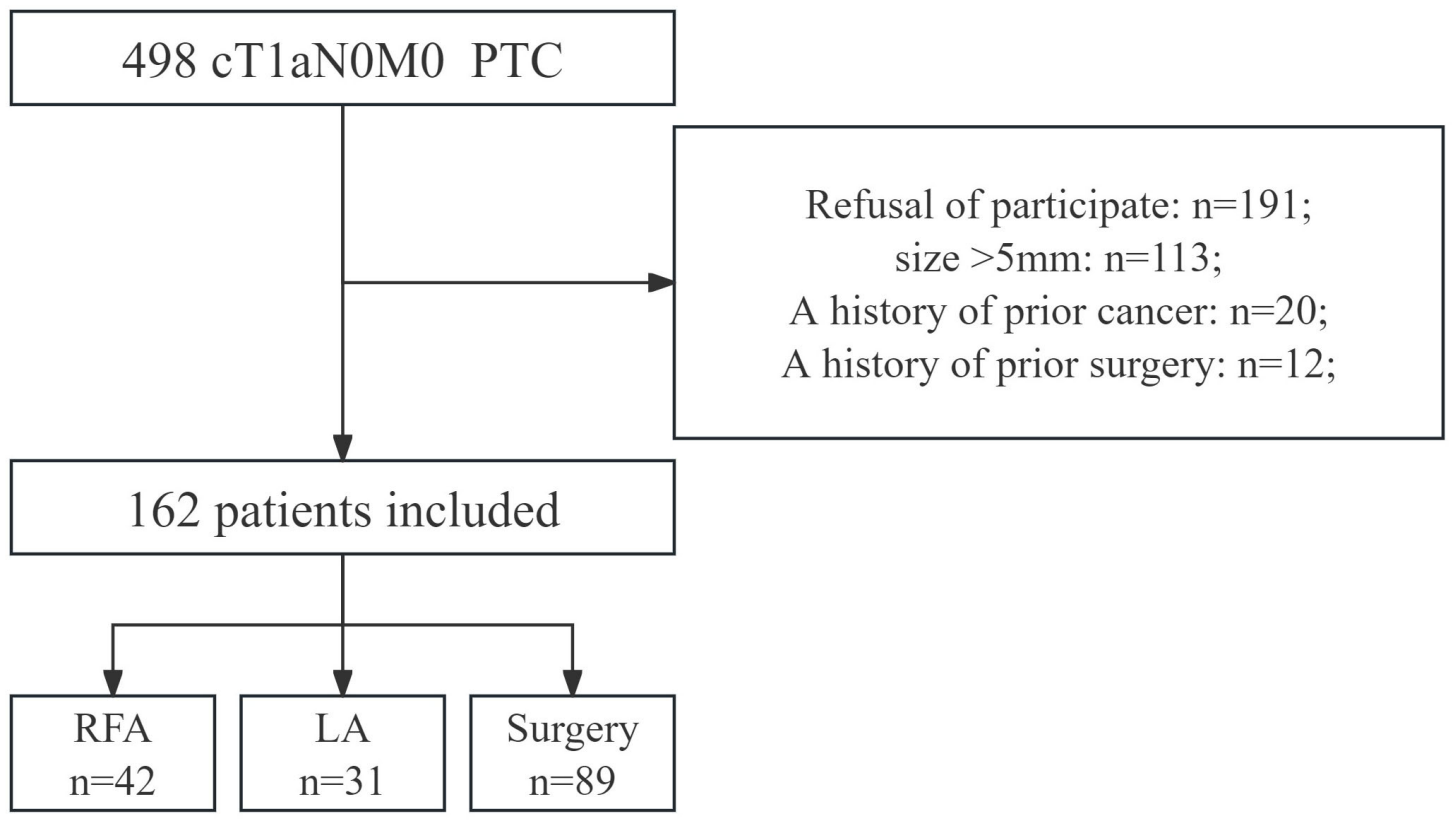
Flow chart of patients enrolled for this research.

Patients who opted for thermal ablation or surgery were included, while those with a history of prior cancer or other specific characteristics were excluded (**Figure 1**). Participants completed the Thyroid Cancer-Specific Quality of Life (THYCA-QoL) questionnaire both before and at 3, 6, and 12 months postoperatively.

### Variable definitions

The absence of clinically positive lymph nodes was defined as cN0 (4). Tumor size was determined by the largest diameter measured via ultrasound (5). Major complications were characterized as those with the potential to be life-threatening, cause significant morbidity or disability, and included complications such as prolonged vocal paralysis (lasting>6 months), neck hematoma necessitating surgical intervention, surgical site infection, lymphatic fistula, and permanent hypoparathyroidism (lasting >6 months) (15). Minor complications encompassed transient voice or vocal dysfunction, transient hypoparathyroidism (lasting<6 months), bleeding managed with local compression, and moderate pain requiring medication (15). Mild side effects such as transient post-procedural pain, heat sensation, and neck discomfort that did not necessitate intervention or prescription medications were not included in the analysis (15). Operation time was defined as the duration between the onset of local anesthesia and completion of ablation in the RFA/LA group, and the duration between skin incision and wound closure in the surgery group (6). Hospitalization period was considered as the time from completion of thermal ablation or surgery to discharge (10). Recurrence was identified as lymph node or distant metastasis (16).

Sonographic characteristics of the nodules were meticulously evaluated, with three orthogonal dimensions of the nodule measured, including the largest diameter and the other two perpendicular dimensions. The volume was calculated using the formula: π * L * W * H/6, where L represents the largest diameter, and W and H are the perpendicular dimensions. The volume reduction ratio (VRR) was calculated as follows: [(initial volume - final volume) * 100]/initial volume. The initial volume was defined as the tumor volume measured on conventional ultrasound before treatment, while the final volume was defined as the volume of the ablated area measured on conventional ultrasound during each follow-up (6).

### Quality of life

The THYCA-QoL questionnaire, primarily designed to assess quality of life post-thyroidectomy, was employed in this study (11, 17). Comprising 24 items evaluating seven scales (neuromuscular, voice, concentration, sympathetic, throat/mouth, psychological, and sensory problems) and six single items (scar, chilly, tingling, weight gain, headache, and anxiety) over the past week. Each item was rated on a four-point response scale from 1 = “not at all” to 4 = “very much”. Scores were linearly transformed to a 0-100 scale, with higher scores indicating greater complaints.

### Treatment

Thermal ablation encompassed RFA and LA procedures, both conducted by the same two physicians (YZ and WG) with 5 years’ experience under local anesthesia. RFA utilized a generator and an 18-gauge internally cooled electrode with 5mm or 7mm active tips (Minimax Medical, China), according to tumor size and surgeon preference. The LA system (Esaote, Italy) included an optical beam-splitting device and a plane-cut optic fiber measuring 1.5 m in length and 300 μm in diameter. During LA, the fiber was connected to a continuous-wave neodymium yttrium-aluminum-garnet (Nd: YAG) laser source operating at a wavelength of 1064 nm with an output power of 3-4 W.

Endoscopic surgery was performed via axilla approaches, while open surgery was conducted through an anterior cervical median incision, involving unilateral thyroidectomy with central lymph node dissection. All surgeries were performed by a professor (SZ) with 20 years’ experience.

### Statistical analysis

During the baseline data comparison, the Chi-square test was employed for categorical variables, while the Student’s t-test was utilized for continuous variables. The primary outcome variables assessed across various groups encompassed efficacy, safety, and QoL. For the evaluation of efficacy and safety, continuous and categorical outcomes were compared using independent-samples t-tests and Chi-square tests, respectively. In the examination of QoL, the outcomes were scrutinized through Two-way repeated-measures ANOVA with the Bonferroni correction method. The threshold for statistical significance was established at p < 0.05.

## Results

### Baseline data

A total of 162 participants were included in the analysis, with a mean age of 50.9 ± 12.5 years, comprising 68 (42.0%) men and 94 (58.0%) women. The average volume of malignant nodules measured 32.3 ± 13.4 mm^3^. Hashimoto’s thyroiditis was present in 17 (10.5%) patients, whereas hypothyroidism was observed in two (1.2%) patients. Central lymph node metastasis was identified in three (3.3%) patients undergoing surgery. These clinicopathologic variables were evenly distributed across all three groups (all p>0.05) (**Table 1**).

**Table 1:** Baseline data of T1aN0M0 patients treated by radiofrequency ablation (RFA), laser ablation (LA) and surgery.

Variable	RFA (n=42)	LA (n=31)	Surgery (n=89)	p
Age	51.4 ± 10.5	52.6 ± 9.3	50.0 ± 14.6	0.286
Sex				
Male	16 (38.1%)	13 (41.9%)	39 (43.8%)	
Female	26 (61.9%)	18 (58.1%)	50 (56.2%)	0.825
Volume (mm^3^)	30.5 ± 10.2	31.8 ± 12.0	33.3 ± 15.4	0.400
Hashimoto's thyroiditis				
Yes	5 (11.9%)	3 (9.7%)	9 (10.1%)	0.940
Hypothyroidism				
Yes	0	0	2 (2.2%)	0.502
Lymph node metastasis	–	–	3	

The symbol "-" represents "no data".

### Efficacy

The mean operation time for the RFA and LA groups was 5.5 ± 4.3 minutes and 6.1 ± 3.9 minutes, respectively, which were significantly shorter than the average operation time of 58.6 ± 23.5 minutes in the surgery group (p<0.001). Patients treated with RFA or LA had an average hospital stay of approximately one day, while those in the surgery group stayed for an average of 4.2 ± 1.3 days, indicating a significant difference (p<0.001). At the last follow-up, complete disappearance was noted in 71.4% of nodules treated with RFA and 71.0% of those subjected to LA. The difference was not statistically significant (p=0.966). There were no instances of cervical lymph node or distant metastasis, nor occurrences of hypothyroidism in the RFA and LA groups (**Table 2**).

**Table 2:** Efficacy of radiofrequency ablation (RFA), laser ablation (LA), and surgery in T1aN0M0 papillary thyroid carcinoma patients at last follow-up.

Variable	RFA (n=42)	LA (n=31)	Surgery (n=89)	p
Operation time (minute)	5.5 ± 4.3	6.1 ± 3.9	58.6 ± 23.5	<0.001
Hospitalization (day)	1.0 ± 0.3	1.0 ± 0.3	4.2 ± 1.3	<0.001
Complete disappearance	30 (71.4%)	22 (71.0%)	–	0.966
Recurrence	0	0	2 (2.2%)	0.502
Hypothyroidism	0	0	–	1.000

The symbol "-" represents "no data".

Over the follow-up period, variations in VRR were observed. In the RFA group, the mean VRR was 26.6% ± 9.4%, 76.4% ± 14.3%, and 93.1% ± 14.2% at 3, 6, and 12 months postoperatively. In the LA group, the mean VRR was 25.8% ± 7.6%, 81.0% ± 10.5%, and 94.0% ± 11.3% at 3, 6, and 12 months postoperatively. Both techniques exhibited comparable changes in VRR.

### Safety

No major complications occurred in the RFA or LA groups; however, four complications were reported in the surgery group (vocal paralysis: n=1; voice change: n=1; hematoma: n=1; surgical site infection: n=1). The difference was not significant among the three groups (p=0.179). Minor complications were more prevalent, with a total of 13 cases. In the RFA group, one patient experienced vocal paralysis and bleeding. Similarly, in the LA group, one patient each reported voice change, bleeding, and pain. Among the surgery group, bleeding (n=3) was the most common issue, while vocal paralysis (n=1) was the least frequent. Nonetheless, the differences were not statistically significant (p=0.733) (**Table 3**).

**Table 3:** Safety of radiofrequency ablation (RFA), laser ablation (LA), and surgery in T1aN0M0 papillary thyroid carcinoma patients.

Complication	RFA (n=42)	LA (n=31)	Surgery (n=89)	p
Major				
Vocal paralysis	0	0	1 (1.1%)	
Voice change	0	0	1 (1.1%)	
Hematoma	0	0	1 (1.1%)	
Infection	0	0	1 (1.1%)	
Fistula	0	0	0	
Total	0	0	4 (4.4%)	0.179
Minor				
Vocal paralysis	1 (2.4%)	0	1 (1.1%)	
Voice change	0	1 (3.2%)	2 (2.2%)	
Bleeding	1(2.4%)	1 (3.2%)	3 (3.3%)	
Pain	0	1 (3.2%)	2 (2.2%)	
Total	2 (4.8%)	3 (9.7%)	8 (9.0%)	0.733

### Quality of life

**Figure 2:**
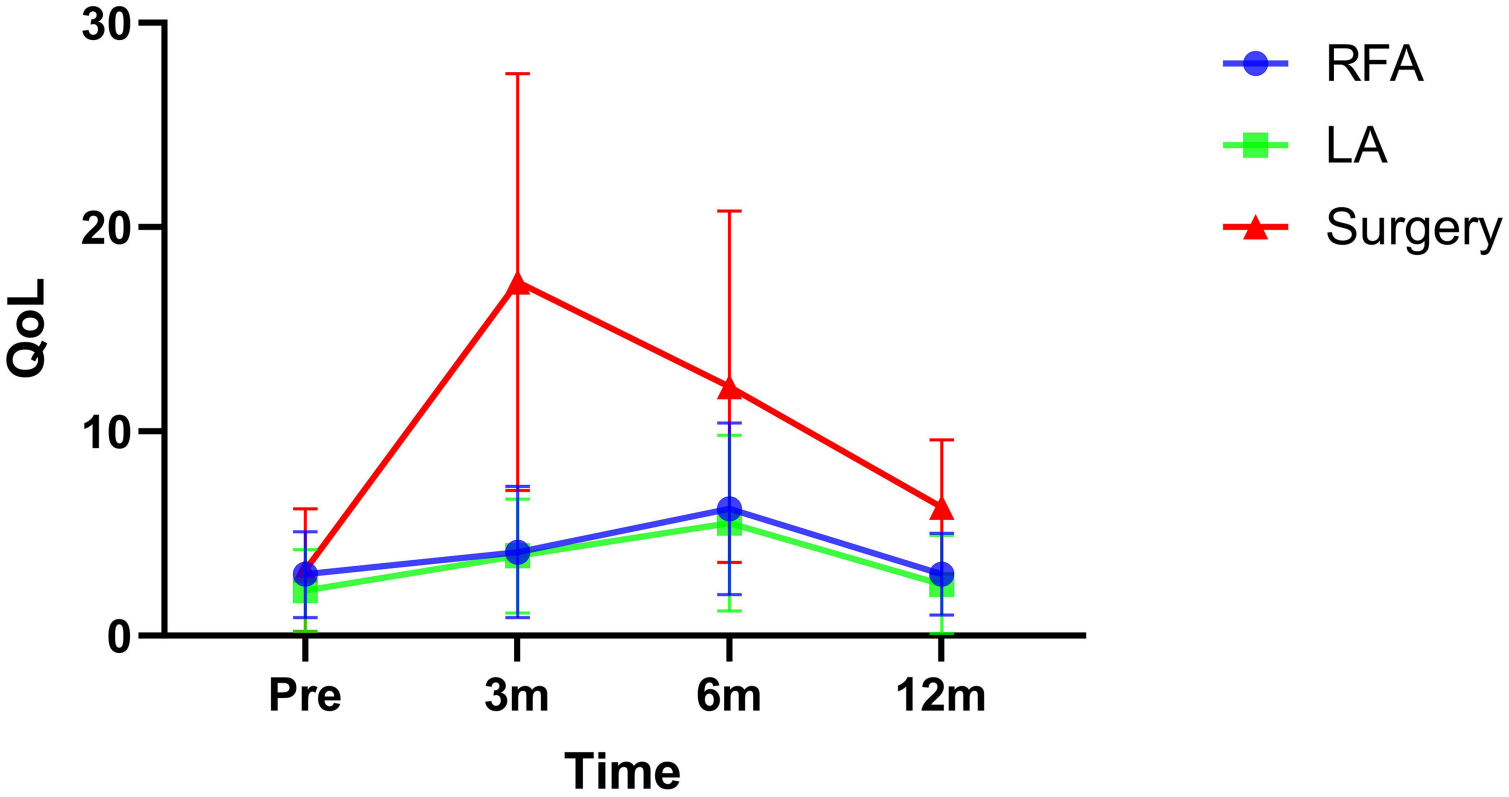
Quality of life (QoL) in radiofrequency ablation (RFA), laser ablation (LA), or surgery groups at different time points.

All participants completed the questionnaire, resulting in a response rate of 100%. Baseline quality of life scores were 3.0 ± 2.1 for the RFA group, 2.2 ± 2.0 for the LA group, and 3.2 ± 3.0 for the surgery group, with no significant differences observed. At 3 months postoperatively, the mean quality of life score increased to 17.3 ± 10.2 in the surgery group, significantly higher (both p<0.001) than those of the other two groups. By 6 months postoperatively, overall quality of life began to improve, with patients treated with surgery achieving a mean score of 12.2 ± 8.6, still higher (both p<0.001) than the scores of 6.2 ± 4.2 in the RFA group and 5.5 ± 4.3 in the LA group. After 12 months, quality of life tended to return to baseline levels, but patients managed with surgery had higher mean scores compared to those treated with RFA (p=0.005) or LA (p=0.004) (**Figure 2**).

### Post-ablation evaluation

At the 12-month follow-up, residual lesions were identified in ultrasound scans for 12 patients treated with RFA and nine patients managed with LA. Fine needle aspiration biopsies were performed on all these cases, confirming the absence of cancer cells, with findings indicating fibrous connective tissue or necrotic cells.

## Discussion

The paramount discovery of our study lies in the establishment of the safety and efficacy of both RFA and LA as treatments for patients with PTC ≤5mm, yielding comparable outcomes with minimal complications. In comparison to surgery, RFA and LA demonstrated equivalent cancer control while posing fewer risks. While the QoL experienced a decline in all three groups following treatment, this decline was less pronounced in thermal ablation procedures than in surgery. Moreover, QoL showed a tendency towards restoration to baseline levels within a year. These results underscore the effectiveness, safety, and advantages of RFA and LA in the management of PTC, providing valuable insights for individualized treatment decision-making.

The issue of safety has been a paramount concern and has undergone meticulous examination. In an extensive review conducted by Ming et al. (18), it was highlighted that a small fraction of patients exhibited symptoms linked to thermal damage. Common complications post thermal ablation in previous studies included transient hoarseness and a burning sensation, with the majority of these symptoms spontaneously resolving within a brief timeframe. Tong et al. (19) also compiled the cumulative incidence of complications observed in RFA and LA, reporting rates of 4.0% for the former and 2.0% for the latter, with no occurrences of life-threatening events. To some extent, our study corroborated these findings, unveiling a complication rate of 4.7% for RFA and 9.7% for LA, slightly surpassing previous documentation. Various factors may contribute to these disparities. Primarily, unlike the studies mentioned, we classified complications as major and minor, with no major complications identified in our analysis. Secondly, the safety of the thermal ablation procedure is intricately linked to the tumor’s location. Tumors situated in close proximity to the trachea or within the posterior thyroid region adjacent to the capsule, where the recurrent laryngeal nerve travels, present a relatively higher risk of recurrent laryngeal nerve injury during ablation. Thirdly, variations in operator expertise and procedural techniques may lead to incidental complications like bleeding and neck swelling. Lastly, inconsistent definitions of complications and our relatively modest sample size may have influenced the outcomes. In the comparison with surgical interventions, while a greater number of adverse events were noted in patients undergoing surgery, the difference lacked statistical significance. This discovery aligns with findings from other reviews (15, 20). Both RFA and LA are minimally invasive methods that induce tissue necrosis through thermal energy, eliminating the necessity for anatomical dissection.

The evaluation of VRR stands as a crucial parameter for assessing the effectiveness of thermal ablation. In a comprehensive analysis by Choi et al. (15), it was reported that the rates of complete disappearance were 48.7% for the LA group and 65.2% for the RFA group. Remarkably, these rates surged to 93% for LA and 81% for RFA in subsequent investigations by Gao et al. (21). These outcomes, including our own, can be attributed to various factors. Initially, the timing of assessing complete disappearance status is pivotal, highlighting the prolonged immune-mediated absorption process of residual tumors within the body. Discrepancies in follow-up durations across available literature may not allow ample time for the complete absorption of certain tumors. Teng et al. (22) showcased a substantial reduction in volume from a median of 55.78 mm^3^ to 0 mm^3^ (p < 0.001) at 60 months post-treatment. Our study aligns with this observation, indicating persistent reduction in tumor size, particularly notable at three to six months post-surgery. Additionally, residual calcification and fibrous scarring within the tumor may impede the complete disappearance of tumor volume. These remnants manifest as non-enhancing zones on contrast-enhanced ultrasound and are confirmed as necrotic material through fine-needle aspiration biopsies (6).

The consideration of QoL emerges as a pivotal aspect that has been relatively understudied in comparative analyses between thermal ablation and surgery. In a prospective study conducted by Zheng et al. (10), wherein 92 patients with PTC underwent thermal ablation and 106 patients opted for surgical excision, both cohorts were monitored for approximately 12 months. Significantly, the surgical group exhibited elevated scores indicative of scar-related issues and heightened anxiety. In the investigation by Li et al. (23), a comparison between the thermal ablation and surgical groups revealed that the former showcased superior ratings in global health, physical well-being, and emotional stability. Notably, except for headaches, the thermal ablation group attained lower scores across other domains in the THYCA-QoL questionnaire when juxtaposed with the surgery cohort. These studies collectively brought to light that thermal ablation was associated with an enhanced QoL relative to surgery, notwithstanding the use of microwave technology in the thermal ablation approach as opposed to RFA and LA. Studies by Lan et al. (24, 25) further reinforced that RFA engendered better QoL outcomes compared to surgery, although their investigations were delimited by a retrospective design. Significantly, the exploration of the correlation between LA and QoL in PTC has been limited in the extant literature. Thus, our study potentially forges ahead by delving into this domain, revealing that both RFA and LA evoke similar effects on QoL, showcasing superior functional outcomes in contrast to surgery. Furthermore, across all groups, the nadir of adverse effects was most pronounced at the 3-month post-treatment juncture, gradually improving towards baseline levels in the thermal ablation cohort as opposed to the surgery cohort. This intriguing finding could potentially be ascribed to the inherently minimally invasive nature of RFA and LA, whereas endoscopic thyroid surgery, while more aesthetically pleasing, triggers the formation of scar tissue within the cavity, a process that initiates healing from the third month onwards.

Previous evidence has validated the favorable outcomes delivered by thermal ablation (1, 3–6). However, all these studies encompassed a large cohort of patients with PTC >5mm in size and overlooked the presence of occult metastasis, a factor significantly influenced by tumor size. In comparison to PTC ≤5mm, PTC >5mm conferred an additional 4-fold risk of lymph node metastasis (13). Although these researchers noted similar cancer control outcomes between thermal ablation and surgery, the detection of metastasized lymph nodes clinically required considerable time, thus limiting the scope of many studies due to relatively short follow-up durations. In our oncology center, the utilization of RFA and LA for PTC was meticulously regulated based on tumor size and other pertinent characteristics. PTC ≤5mm presented an exceedingly low risk of occult metastasis.

There is a divergence in the management of small, low-risk PTC globally. While active surveillance is deemed appropriate for patients with small, intrathyroidal PTC lacking aggressive cytopathology, local invasion, or clinically detectable metastases, and has shown favorable outcomes such as a low growth rate, rare regional or distal lymph node metastases, and low mortality (26, 27), Chinese patients often experience anxiety and nervousness due to the potential diagnosis of even a small PTC. As a result, immediate treatment is typically sought based on the psychological state of the patient, and preoperative fine needle biopsy is mandated before definitive therapy according to official guidelines in China (28, 29). On the other hand, long-term QoL considerations are paramount in the management of indolent, slow-growing PTC, particularly in the current era of conservative management. Options have expanded to include minimally invasive approaches; however, unlike active surveillance, any invasive therapy could potentially diminish QoL to varying degrees. Therefore, the burden of surveillance and the level of concern should be carefully weighed when determining the most suitable approach (30).

Acknowledgment of the limitations of the current study is crucial. Firstly, the relatively modest sample size may have reduced the statistical power of our findings, highlighting the need for a larger size trial. Secondly, our inclusion was limited to patients with PTC ≤ 5mm in size, necessitating future studies to incorporate a wider range of PTC sizes. Lastly, as this study was conducted at a single institution, external validation is essential before widespread clinical implementation.

In conclusion, for PTC ≤5mm, both RFA and LA exhibited similar cancer control outcomes and superior quality of life on par with surgery, while minimizing complications. These findings underscore the promise of RFA and LA as potential standard treatments for small PTCs, subject to further confirmation in future studies.

## Data availability statement

The original contributions presented in the study are included in the article/supplementary material. Further inquiries can be directed to the corresponding author.

## Ethics statement

The Henan Cancer Hospital Institutional Research Committee approved this study, and written informed consent agreements for medical research were obtained from all patients before undergoing initial treatment. The studies were conducted in accordance with the local legislation and institutional requirements. The participants provided their written informed consent to participate in this study.

## Author contributions

WG: Writing – original draft, Writing – review & editing. RZ: Writing – original draft, Writing – review & editing. SZ: Writing – original draft, Writing – review & editing. YZ: Writing – original draft, Writing – review & editing. CZ: Writing – original draft, Writing – review & editing. DZ: Writing – original draft, Writing – review & editing.
